# Practical knowledge of experienced nurses in critical care: a qualitative study of their narratives

**DOI:** 10.1186/1472-6920-14-173

**Published:** 2014-08-18

**Authors:** María Sagrario Acebedo-Urdiales, José Luis Medina-Noya, Carme Ferré-Grau

**Affiliations:** 1 Rovira i Virgili University, Av. Catalunya, 35 CP43002 Tarragona, Spain; 2 University of Barcelona, Barcelona, Spain

**Keywords:** Practical knowledge, Practical wisdom, Nursing, Critical care, Clinical experience

## Abstract

**Background:**

Scholars of nursing practices have claimed *practical knowledge* is source of knowledge in its own right, nevertheless we know little about this knowledge associated with day-to-day practice. The purpose of this study is to describe knowledge that the more experienced nurses the in ICU make use of and discover the components of care it includes. Understanding this *knowledge* can contribute to improving the working practices of nurses with less experience.

**Methods:**

We used a phenomenologic and hermeneutic approach to conduct a qualitative study. Open in-depth dialogue interviews were conducted with 13 experienced ICU nurses selected by intentional sampling. Data was compiled on significant stories of their practice. The data analysis enabled units of meaning to be categorised and grouped into topics regarding everyday practical knowledge.

**Results:**

Knowledge related to everyday practice was evaluated and grouped into seven topics corresponding to how the ICU nurses understand their patient care: *1) Connecting with, calming and situating patients who cannot communicate; 2) Situating and providing relief to patients in transitions of mechanical respiration and non-invasive ventilation; 3) Providing reassurance and guaranteeing the safety of immobilised patients; 4) The “connection” with patients in comas; 5) Taking care of the body; 6) The transition from saving life to palliative care; and 7) How to protect and defend the patient from errors.* The components of caretaking that guarantee success include: the calm, care and affection with which they do things; the time devoted to understanding, situating and comforting patients and families; and the commitment they take on with new staff and doctors for the benefit of the patient.

**Conclusions:**

These results show that stories of experiences describe a contextual practical knowledge that the more experienced nurses develop as a natural and spontaneous response. In critical patients the application of everyday practical knowledge greatly influences their well-being. In those cases in which the nurses describe how they have protected the patients from error, this practical knowledge can mean the difference between life and death. The study highlights the need to manage practical knowledge and undertake further research. The study is useful in keeping clinical practice up-to-date.

## Background

In their attention to people in critical care, clinical staff faces one of the greatest professional challenges. Caring for critical patients requires nurses to take numerous decisions. Their practices are part of the challenge of maintaining care in a highly technological environment. They are everyday practices that, when performed by experienced nurses, are saturated with *know-how* that is classed by its scholars as *practical knowledge*[1].

The concern for distinguishing the best knowledge to use in practice has been a constant presence in the field of nursing care [2-4]. Carper [5] in his study on the knowledge used by the nurses already indicates that because of its very nature, the practical knowledge has to come from many sources and proposed a model beyond empirical knowledge. Benner, in his studies on the levels of competence, and the clinical and ethical judgement of the expert nurses indicate that practice is a source of knowledge in its own right because in practice, knowledge is not only used, it is also developed [6,7]. In addition, experience-based knowledge is indicated as one of the categories that the nurses attributed to their sources of knowledge in practice [8].

Nevertheless the practice of caretaking and the knowledge and experience used while doing so, as is topical, is characterised by being invisible and difficult to quantify since a significant part of nursing care is relational and therefore difficult to identify and recognise [9-12]. The question that arises from this problem is: How can we study this?

Therefore, we asked ourselves how the practical knowledge of intensive care nurses can be identified and evaluated.

In critical care nursing practice, there is strong pressure that the best evidence is firmly linked to the positivist paradigm. Nevertheless, its suitability for research into nursing care as something that can be measured and rigorously applied has been questioned on numerous occasions [13,10,14]. Closs, and Cheater [15] have already considered that there is an interaction of multiple factors that affect the decisions regarding patient care, and that this type of research, although necessary, usually only provides a partial image, and more detailed and contextual information derived from other types of research or consensus is needed. The variety of the forms of both technical and human knowledge used by the intensive care nurses, which are difficult to articulate, lead to the need for further research with a more practical focus, recognising that different types of problems require different methods of study [3,16].

There is, in addition, a current of sociocritical thought that attempts to use classic philosophy to restore practical knowledge, *phronesis*, which deals with human judgements and values and indicates that this is the nature of nursing practice. *Phronesis*, defined as unique knowledge that determines the specific actions in each situation, requires investigation into the practical nursing action that is found within the framework of the interpretational and sociocritical paradigms [17,18,10]. The practical knowledge, in addition, like any other form of social action, can only be understood within the context in which it takes place [19].

These ideas are congruent with the statement by Closs, and Cheater [15] that practice based on the best evidence is not simply a pragmatic logical process, that involves the access and subsequent use of the best research data, but rather an interaction of multiple factors that affect the nursing decisions regarding patient care. It also concurs with the thoughts of Benner on *practical knowledge* as wisdom that does not come from an intellectual source, but is the product of the experiences that the professional has accumulated over time and how he or she has been involved in it [18].

In line with this thought, *practical knowledge* is complex and is not certain knowledge [20] and intuitive [21] but it is revealed in the voices of the nurses as know-how that is a source of satisfaction in their work, shown in the continuous improvement of their practices [1,11,22,23]. The narrative focus represents a chance to explore the reflective way in which nurses approach the different dimensions of caregiving and how they build their identity on the stories they tell [24,25].

The literature review shows that few studies have been carried out on practical nursing knowledge and that the practical aspect must be investigated using methods that can capture the details of caring, because practical knowledge is essentially emotional and intuitive. In the nursing profession this practical knowledge, although based on formal knowledge, is learned and constructed through experience.

The purpose of this paper was to study the practical knowledge of ICU nurses as expressed in their narratives. The question that has guided our research was: What practical knowledge do the ICU nurses describe? This paper identifies and analyses the emerging areas of knowledge and explores the components of caring in how they implement their practice.

## Method

### Design

It is a qualitative study, inspired by phenomenology and hermeneutic philosophy for interpreting the *know-how* that the nurses describe in significant stories of their practices in ICU patient care. The research method used complies with the Qualitative research review guidelines – RATS.

### Scenario and participants

The scope of the study is limited to intensive care units for adults. The research took place in six ICUs in Catalonia (Spain). Thirteen nurses – all women – took part in the study. The male/female ratio in the ICUs the participants are from is approximately 1/20.

The criteria used to select the *professionals with experience* were that they had to have 1) more than five years of experience in intensive care units; and 2) gained the respect of their colleagues for their good practices. The criterion of more than five years of experience in the unit also relates to the levels of competence described by Benner [6], who considers five years to be the minimum time necessary working in the same unit to have acquired the skills that characterise expert practice. The second criterion – that of having earned the respect of their colleagues for their good practices – reflects Gadamer’s *concept of experience*[26], and is the result of questioning preconceived notions. It is not theoretical or technical knowledge, but rather that true experience is characterised negatively, in the sense that we acquire experience on something when we realise that it is not what we thought and that after the experience we know the object better. In nursing these ideas have been created in research by Benner in a belief that proclaims clinical skills and the underlying practical knowledge to be capabilities acquired with the passage of time as a product of history and the way the professional is involved in it [1,7,27]. This experience in the case of the expert nurses has enabled them to acquire a specific, tacit, intuitive and ethical knowledge. To select the nurses with the skills required, their colleagues were asked the following question: *“If you had a close relative (child, partner, parent, etc.) in the ICU, who would you like to take care of them?”* This question has a clear affective component and was used as a resource to select nurses with high levels of competency, in both the technical and relational sides of caregiving.

### Data gathering

The data was gathered through open interviews with in-depth dialogue. The question used to start the conversation was: *"Can you tell me a story about your work in the ICU that you particularly remember?"* The idea that led us to look at this question, in line with the thinking of Benner [24,7], is that the stories of the practices that the nurses remember are those that are significant in the *present*. We assume that these memories are important moments so they represent learning or reaffirmation of practical knowledge that illustrates how the nurse perceives, feels and acts. After the initial question, we attempted to continue the interview with the minimum interruption possible by the interviewer, only what was necessary to clarify meaning or particular details. The most common questions that were asked to identify practical knowledge were: What did that particular situation require? What were your priorities? When you reviewed the situation, what prompted you to act in the way you did? The duration of each interview was between one and two hours. The interviews were recorded and transcribed afterwards.

### Rigor

The data has been interpreted by the three researchers in the study. With a view to credibility and confidence, we proceeded with the aim of comprehending the details and the entirety [26]. To guarantee the clarity and credibility [28], the nurses involved were asked about the nature and the meaning of the ideas expressed in the details and scenes of their narratives and this was reinforced with the comments of other researchers. The transferability was undertaken using direct quotes in the presentation of the results (N: Nurse, YE: Years of experience in the ICU).

### Analytical procedure and data comprehension

In the data analysis we turned to the segmentation and identification of the main subjects in a circular and dialectic process [29,30]. Each interview was read several times to identify stories and issues, and to obtain a global vision of the tale. Then the categories were established, in which previously constructed systems were not used, so it can be said that it was predominantly an inductive process. Finally, day-to-day practical knowledge in patient care that integrates the results obtained was identified and conceptualised.

### Ethical considerations

The data have been treated in accordance with Spanish Organic Law 15/1999, of December 13, Protection of Personal Data [31] the Law 14/2007 of 3 July, on Biomedical Research [32] and the Declaration of Helsinki of 2013.The study was approved by the research ethics committee of the Gimbernat University Schools of Nursing (the Escoles Universitàries d’Infermeria Gimbernat). All of the participants received oral and written information about the study. This study involved no risk for the participants. To meet the criteria of permission and confidentiality the informed consent of the participants was requested and, upon acceptance, the interview was recorded. Anonymity was maintained at all times when handling the data, the names of the patients and professionals that appear in the transcription of the stories have been changed.

### Limitations of the study

This study has the limitations inherent to methods of qualitative analysis. The participants, nurses, expressed diverse experiences and are possible that not all kind of practical knowledge had been captured. In the context of this study the data is of considerable social and medical value, but cannot be generalised to services with different sociodemographic and cultural characteristics.

## Results

Overall our results show that the nurses with the most years of experience in the critical patient care in the ICU have developed a tacit and intuitive knowledge that is shown in the detailed description they give of their everyday practices. Specifically, the everyday practical knowledge that the nurses have described have been grouped into seven topics. The first six correspond to direct patient care: 1) Connect with, calm and orient patients who cannot communicate; 2) Situate and provide relief to patients in transitions of mechanical respiration and non-invasive ventilation; 3) Provide reassurance and guarantee the safety of immobilised patients; 4) The “connection” with patient in coma; 5) Taking care of the body.; 6) Patient support in the transition of care from saving life to palliative care. The final group deals with the implication and commitment to the other team members in the things they do well, which we have named: 7) How to protect and defend the patient from errors.

### Connect with, calm and orient patients who cannot communicate

Four of the nurses studied describe how they communicate with patients that have lost the ability to speak and ask for what they need. These are patients with facial palsy, intubated patients with little sedation that are kept awake or patients with tracheotomies. This is *know-how* that the expert nurses base on the recognition of typical response patterns, on the fear that the patients express of not being understood and on the fears they anticipate.

*“… it's very hard to understand him because he has facial palsy, he has some mobility in his hands but this is quite limited… he's awake, he's distressed, he wants to speak, to communicate and there many impediments to communication… we've got some cards with the most common things that might happen to them, if they are in pain, if they want to change position, if they want to know something. … so he points to what he wants. Sometimes we write the alphabet on a whiteboard and they point … I try to ask again, because sometimes you go to the most basic thing and it may well be some other worry and so I say 'let's start again' … above all I believe they worry about their condition and how long it might all take… I try not to lie to them, it's my job, but it's the doctor who's responsible for explaining everything to them: it's explained to them but I don't know up to what point they do or don't understand, the medical parts are very technical, so I try to tell them the truth that it's a long process”,* (N4/ YE: 18)

In this tale we can see how the nurse also feels it is important to respond to the patients honestly, putting them in a position where they have an accurate idea of what to expect at all times. Although sometimes it seems they feel the load is excessive and they are tempted to leave, “*I try not to lie to them, it's my job, but it's the doctor who's responsible for explaining everything to them…” their* interest in understanding the patient and their effort to respond to what they need is evident.

This interest in ensuring the patient is connected also leads the nurses to ask the family to bring in objects from their daily life that can link them with their world:

*“…we've asked the family to bring him a radio, because he's a gentleman who likes this a lot and it'll also help him to have more contact with the outside world… We've also asked them to bring in his aftershave… to help him remember himself and his life”,* (N 4/ YE: 18)

The know-how of understanding the patient is also show in patients with tracheotomies. The following story shows details of day-to-day practice performed with energy and a sense of humour. The nurse shows extraordinary patience in connecting with the patient and attention given with care and affection, without forgetting to be watchful of the risks, and whose results produce satisfaction:

*“…with trach patients… I spend longer looking at how they vocalise, to understand them… I don't go until I have understood them, and it drives me mad when they give up… 'Again! So what if I don't understand, it doesn't matter. You have to get used to me and I to you'…. every person vocalises differently and you have to get used to it, and when you've spent a few days with them you understand them and know what they want… Content, satisfied, with a smile, and if not, well sometimes you just don't manage. It brings a lot of satisfaction when they say I understand them, that I follow them with my gaze, you have to look at their faces you have to keep trying… We say to them 'If you don't communicate… We'll do what we want with you; there are a lot of women here'… (Laughs)… start putting your radio on, but never with earphones, because if something happens we'll all hear it”,* (N 9/ YE: 20)

This nurse also describes how to act with patients that have suffered a trauma. The nurse's main concern is to make them aware of their situation, explaining why they have got a tube, how they are and what they can expect:

*“… For trauma patients it's like being in a dream, they were walking down the street and you have to remind them why they can't speak… and of course they are scared. I start from scratch, I explain why they've got the tube,… that in 24 hours it'll be closed, that it's temporary. Sometimes they listen and sometimes they don't pay attention, they think they're dreaming that it's a nightmare… Gradually they start to see the situation, you have to keep telling them”* (N 9/ YE: 20)

Another nurse narrates in great detail about their interpretation of experiences with *agitated patients*. They also show how they create distraction strategies guided by intuition, that go as far as “*going along with*” patients who have hallucinations, in an attempt to get into the world in which they are living, which is their reality at that time:

*“…the way I speak to them, my voice calms them down, I distract them, I speak to them, I explain things to them, I let them know where they are, and that calms them, I hardly ever have to medicate them to sedate them… for example when they have hallucinations I have an extremely good ability to go along with them, for example if they see flies I say: “I'll get the fly spray out and we'll kill them, it's OK, we'll close the door so they won't get in again… things people don't think of. We have to remember that the patient who tells us these things is living them as if they were real, I have a lot of ways of calming them…”,* (N 6/ YE: 29)

Although it is not the majority of nurses who tackle this issue, those who do are professionals with many years of experience. Their description and intuitive reasoning occupies a significant part of the story and it contains great wealth of details.

### Situating and providing relief to patients in transitions of mechanical respiration and non-invasive ventilation

This topic is approached by six nurses. In the transition to mechanical ventilation, what the nurses are interested in is calming patients' anxiety (due to lack of air) and not increasing it. When the decision to intubate is taken, the nurses describe reassuring the patient as priority. Sometimes, as related in the story, they do not overload them with details about placing the tube and only provide them with the information that they are going to be sedated and then they'll breathe more easily, which calms them. Other times, like with a patients with progressive paralysis, they do not want to deceive the patient, although they do not use the word intubation, and then explaining the situation to the patient afterwards.

*“…What we try to do is reassure, saying 'Now we're going to send you to sleep and you'll see how much easier it'll be to breathe, … now you're going to have a rest, we're going to send you to sleep', but actually telling them you're going to intubate them levels them, but the important thing is that they are going to sleep and they'll be able to breathe better”* (N 5/ YE: 7). *“… I explained that they were going to sedate him and that he wouldn't feel any pain and would be comfortable. So despite his concerns he was calmer…. after the intubation where they are, if they can't remember, what the situation is… suddenly they wake up with the tube, the probe, lots of catheters, so we explain a bit about what's happening and try to keep them pain free… not deceiving them is important too, they're not idiots, they're patients, so I explain to them that it's not a process that will be completed in a day and that they will be fine, but it'll take time, in a positive way, explaining that they're OK now, … but that it's a long process and they're going to be here for a while”,* (N 4/ YE: 18)

The way both stories coincide in avoiding the explicit information on the intubation gives rise to the doubt as to whether this is a product of their experience that they were taught it is best not to anticipate because, as the nurse said, “*it levels them,”* or a strategy for not having to deal with the patient's fears.

In the withdrawal of mechanical ventilation the nurses show a clear knowledge of the relationships that make it easier. They describe in detail, making qualitative distinctions, the attitudes they maintain and the help that makes it easier for the patient to be successfully removed from mechanical ventilation. They show they are aware of the value of nursing work and what it means to be at the patient's side in this situation, of the importance being calm and fully focused on the patient for the success of weaning. Two nurses tell us of this:

*“…when we are weaning a patient… depending on the professionals that are there, the patient is more likely to get through it or have to be intubated again. If you are calm, if you don't get anxious, if you don't get stressed, if you know how to evaluate the situation the patient might not have to be reintubated, but if you're overloaded with work, you get anxious and start to shout, the patient is gets worked up and will probably need to be intubated again. … The stage of withdrawing the machine and taking out the tube, if you are not ready to do it, it might fail and the patient is intubated again, it's a step back… there are some steps you have to follow, but if you are there with him and you follow them with him, that patient will get through it …”* (N 6/ YE: 29). *“…very agitated…he struggled with the respirator…I thought maybe he didn't know where he wass…I explained everything, that he had had an accident, he'd broken his leg, his arm, some ribs… I told him he'd been here 20 days… that he was connected to a machine that had been helping him breathe but as he was better they were trying to take him off it, but if he… wasn't calm we couldn't take him off it… I went to speak with the family… then they came in and explained it to him too… the following night when I went back they had already been able to extubate him…”* (N 11/ YE: 8)

Three nurses describe the reasoning and action with the patient with non-invasive ventilation. These nurses tackle how to situate and comfort a patient wearing a mask. It is a practice that aims to relieve the physical and emotional discomfort caused by a lack of air and the oppressive sensation of the mask, and overcome the communication difficulties that the patient has. Some methods that the nurses describe in this practice are: being there, giving them time to trust the procedure, say what is causing discomfort or what they need and avoiding a lack of synchronisation; relieving areas of pressure; reassuring them it is temporary; and agreeing periods of rest:

*“…one very important thing is clearing the cubicle …..you have to stay with him… explain the steps that are going to be taken, what he will feel… first you will feel a sensation of suffocation… let the machine take you, I'll be there the whole time, if you touch it'll leak… and then it won't have the effect we want*…”(N 12/ YE: 6). *“…generally it stresses everyone because later they say they are OK because they can get much more air and when you take it off, I mean when it's put on, it makes them very anxious in this respect because they can't speak and that's what panics them the most, because of course you can't understand them, what with the oxygen tube, the mask properly tightened, they can't speak, so this makes them more anxious as they aren't able to communicate with you… calming them above all, 'we know you can't speak now, …, 'when they take it off you can tell me what you want to say,' because it'll be half an hour or an hour and if you see it's not possible you try to approach them so they speak and you try to understand what they're saying”,* (N 5/ YE: 7). *“…explaining first … 'Look, I know this is going to be hard but we're going to give you breaks… if you can't handle it after ten minutes we'll take it off' very short periods, not that we'll take it off after four hours, so he would think, 'nobody would put up with this for four hours,' ten minutes yes, and you go back, you go back to the place, 'How are you?' and I take it off… and maybe while you've got the ventilation off you moisten his lips, you let him rinse his mouth with chlorhexidine, even sometimes let him chew gum… if you apply colloid plates in the areas of pressure before you put the mask on you avoid ulcers forming in these areas… when you secure the mask do it with the minimum pressure to make up the volumes, the minimum… I think it is all about spending time at the beginning and then agreeing with the patient that you'll be there, and then actually being there…”,* (N 2/ YE: 20)

In the tales of these practices two common characteristics stand out in the nurses: the knowledge of the typical response models that show how they anticipate the patients; and the calm and precise manner in which they do this, which can be seen in how they describe the need to focus on the patient and the importance they give to situating and providing relief to the patient throughout the process. These descriptions show the complexity that knowing the patient and tranquility entail as components of caretaking.

### Provide reassurance and guarantee the safety of immobilised patients

Four nurses tell us how they provide safety and reassurance to immobilised patients. In the following paragraph the nurse tells us, as if she were there, the interpretation of the fears and how to act to achieve the greatest emotional well-being and increase the sensation of control of a young patient with a spinal cord injury frightened of being moved using a hoist. And as she calms him, backed by her experience, she is watchful of the interpretations he may make of what he is hearing, without overlooking action needed to avoid risks:

*“…'I promise you won't fall out at all, never, this hoist is so safe, I have never in my life seen anyone fall out of this hoist; I've been here for 20 years and I've never had a patient fall out of a hoist, you'll be the first if you do, the first, right… hook the hoist at the distal side, now hook the hoist at the proximal side,' then the other says 'good' then I might look and see that they do it like that, but they mustn't see you're unsure, don't say, 'Ooh, it looks like you twist it, right?' it's silly but not 'It looks like you twist it here,' not, 'You twist it, right?' Do it like that and change it, but you don't tell them, make them feel safe…”* (N 2/ YE: 20).

A nurse describes her reasoning and recounts how she acts with immobilised patients who are not aware of what has happened. Her main concern is to make them aware of the situation, explaining why they cannot move or how they are and what they can expect:

“…admitted 4 days ago due to paralysis, the origin was not clear and appeared to be Guillain-Barré syndrome. … the first two days he was conscious and then he had to be intubated… As soon as he wakes he's going to be more and more anxious each time because he'll see he can't move, he'll see he has a tube, the techniques, and if he can't sleep he'll have hours and hours with it going round his head… I usually start by explaining the situation to them (N 4/ YE: 18).

The following stories demonstrate the nurses' concern when they have to immobilise a patient. This is a practice that presents two very different aspects: the first is the ethical dimensions due to the patient's limited autonomy, and the second relates to taking care that they do not fall or extubate themselves. In the first story, although the words “*I'm going to remove his restraints, it's a promise I make them*” indicates a concern for what it implies for the patient to be immobilised, she clearly states that the priority is to guarantee the patient's safety. In the second there are indications of ethical considerations in the need to justify the restraint for the patient's safety, in their interest in explaining this to the family so that they understand, and in the way they categorically state, “*We are always defending the patients*”:

*“… if someone lowers the guard rail and doesn't put it up again afterwards, I follow it up, even if it's the head of the department. The guard rail always has to be up, as they run the risk extubating themselves and that's a step back in the process,… In sedation, the moment we're going to remove the tube, I'll remove the restraints, it's a promise I make them…”* (N 9/YE: 20). *“…we are always defending the patients… if we believe we have to restrain the patients we will always restrain them for their safety, more importantly, if it's in the extubation period and they can't be given anxiolytics or sedatives. If you explain to the family why you haven't removed the tubes they understand perfectly.”* (N 7/ YE: 21)

The nurses that deal with this practice stand out for their years of experience and their commitment to protecting immobilised patients.

### The “connection” with patient in coma

Five nurses talk about the connection with coma patients. They are stories in which the nurses describe how to speak with patients in comas in a certain tone of voice, how they introduce themselves and how they tell them where they are, what has happened, what they are doing at all times and any information that may help them know where they are. Sometimes they stress it is good to touch them a lot, convinced of the positive result of this practice and they specify that when patients in comas have woken they have memories of their voice and of the reassurance that their presence transmitted:

*“…There are a lot of patients that have come out of a coma or from long periods of sedation that when they woke up they knew who I was, they tell me they remember my voice, the tone of my voice. Of all the things I do, one is to introduce myself to the patients, whether they are awake, sedated or in a coma, I touch them a lot, they tell me that they remember my tone of voice, the way I spoke to them, how it calmed them because they knew who was coming to work this shift. … I touch them, I speak to them, I introduce myself, I explain everything I'm doing to them, “Now I'm going to move your legs” now I'm going to tickle you…, now I'm going to give you a massage…”,* (N 6/ YE: 29) *· … although they are sedated and sleeping, you always have to talk to them and touch them… even if you don't speak to them, simply touching them shows them you're there and if they need anything you're there, they're not alone”* (N 11/ YE: 8) *“…I mean that girl was sedated…? So then this girl at eight in the morning explained to me that she knew she'd had an operation… in other words everything… I remember that she said there was someone who'd said, 'shut up,' right? She knew that people had come in, that they'd spoken to her… I explained to her what I knew and what I didn't… and so you say.... we've still got a lot to learn” (*N 8/ YE: 20)

In the following stories, the nurses, although they say they do not believe the patients can hear their voice, it is a practice they say they follow “just in case”. In the first of the stories, the nurse associates it with the changes detected in the patient's vital signs when there is an emotional event (like when a sedated pregnant patient hears the baby, or when a relative is present) and the lack of knowledge we still have of sensory perception in sedated patients:

*“…We had a 21-year-old girl with an aneurysm… I spoke to her a lot, I don't know. I paid her a lot of attention. … and then I told her, I don't know, that she had to fight, that her family was very anxious outside, that her boyfriend hadn't left for a moment,… (Why did you tell her this?) Just in case… I don't think so, but just in case. In case she can. We don't know… I've only seen it with a patient who was pregnant…”… I like dealing with these patients, I don't mind being there for seven hours without moving and when they hooked her up to hear the baby, to listen to it's heart, she went into tachycardia and she was very sedated…at times the patient is sedated and when the family comes in, you see the patient is experiencing tachycardia, is anxious, and moves …What you say to the family is that when they are there they should touch them, we don't know what they can hear, which they can't hear, they don't remember anything, few patients remember, you have to speak to them, touch them, tell them things.* (N 9 / YE: 20) *“…is speak to them out of habit, but with little hope that… but you keep doing it anyway…”* (N13/ YE: 7).

It is notable that the majority of the nurses that tackle this issue with a wealth of details are professionals with many years of experience and this gives us an idea of the significance of this accumulated experience. All of the descriptions show how these nurses value comforting and situating as fundamental components of their care. This idea is reinforced by the fact that the nurses, although not convinced they are being heard, continue to do this.

### Taking care of the body

Knowing how to take care of an injured body and prevent sores is a value underlined in the nurses' stories. It is a practice that is present in nine stories. This is precise care that takes into account details of how to make the bed when it is not possible to move the patients, how to wash them so they are comfortable and not increase their ICP (intracranial pressure) or how to lessen the perception of fear of suffering further harm. This is how this nurse describes it:

*“… You straighten the sheets often, you try to harmonise the bed, palm up, palm… I went in, and not to move them, to massage them, then although you might not be able to change the sheets, you already are already touching if they've got anything on their back, change their point of support… if there is anything that shouldn't be there, you straighten the sheets, we do what we call the four corners, four tensed and then you know there are no wrinkles in the bed, you play around raising and lowering the bed, so as not to have the same point of support all the time, palm up palm down… they are calmer, they always are always grateful, not at the time, but afterwards they are… we move them, because if they are in pain that also increases their ICP and we sedate them a little more and we move them and that's it and it's good for most of them, I've never seen anyone die from being moved or changed but I have seen people end up bad for not having done it”,* (N 9/ YE: 20).

The nurses tell of the fear of injury that they perceive in the patients and what they do to “distract” their attention with other aspects of caregiving. And they demonstrate the involvement and commitment they transmit to the patient using the first person plural *“it's very easy for us to get ulcers”* when speaking of the significance of taking care of the body:

*“…a patient that doesn't want to let us wash, lift or do anything to him or her, 'Well, let's see. Good morning, now we're going to see this gentleman, we're here to give him a wash and take advantage of that to check his back, here in this unit it's very important because it's very easy for us to get ulcers,' you have just made the wash a much smaller issue and put it to one side as a problem and made it into a way to see other very important things, 'it's not the soap and water, we could do without them, but letting me see what's behind is really important,' so I don't give them time to say yes or no, I don't let them doubt, or give them arguments”,* (N 2/ YE20). *“I made her participate as much as possible to hurt her as little as possible, and when she said that was enough, I listened to her and stopped.”* (N 1/ YE: 5)

Knowledge that is also evident in how they deal with comfort and preventing or relieving pain by caring for the body and the significance in this practice of touching, mobilising, massaging or respecting sleep:

*“…Physical contact is very important, because you have to touch the patients, you have to scratch their ear, you have to massage their legs, because they can't move, basic things they would do if they could …”* (N 6/ YE: 29). *“….be in a straight position in bed, keeping the bed clean… at night for example respecting sequences of wakefulness and sleep … you can go into the cubicle and not turn then light on or just turn on the emergency light … keep this in mind a bit” …”* (N 10/ YE: 10). *“…we try to make changes at the patient's request, because for example he is in a lot of pain, but it is a pain that is not relieved with painkillers, it is relieved by changing his position more than with the painkiller he's given”.* (N 4/ YE: 18)

In the story of these nurses some common characteristics are evident in their reasoning and how they do things provides an idea of the value they link to this practice: the time devoted to ensuring the patient's well-being and the touch and affection their practices transmit.

### The transition of care from saving life to palliative care

Seven nurses tackled the issue of the transition of care from saving life to palliative care. In these stories it is shown that among these nurses there is a consensus that the transition to palliative care must lead to a dignified death that is above all painless, in which priority is given to the patient's comfort, and the possibility of saying goodbye to their family and they find it frustrating when patients and families are not given a realistic picture of the situation:

*“…everything was to comfort her, we were watchful that she wasn't in pain, giving her pillows… It was delicate work”* (N 3/ YE: 20*). “…that although she was dying, she could be calm, and have a dignified death…that she wouldn't die alone…Nobody, nobody was straight with her”* (N 1/ YE: 5). *“… is more aimed at comfort…”* (N 12/ YE: 6).

Nevertheless in the way they approach the practice, there are a lot of differences that depend, fundamentally, on whether they are quick or slow routes.

When the route is quick and above all when there is the expectation of survival by everyone (patient, family and team), the death occurs without time to approach the transition with the patient and with the suffering and frustration for the unexpected that is seen in expressions such as *“then well, anyway”*, the only care that seems possible is sharing the despondency with the family:

*“…From when he really became ill until he died… there was very little time… And when they came, well, they came in quickly, they hugged him and stayed there at his side for ages.… We let them say goodbye to him… Then, well, anyway, we were there with the family…”, (*N 5/ YE: 7)

In other situations, where things move more slowly and there is time to adjust the care priorities, the nurses describe how they change actions that would focus on saving life to care that seeks comfort. A good example is found in the following story, in which the nurse explains how she changes her priorities and how step by step she adapts her response to what she considers important for the patient's well-being:

*“…It is very progressive, normally it is people who are there some time and you gradually realise through impairment accompanied by clinical criteria. You realise that things that seemed very important to me no longer were, and other things started to be more so… When I have a patient it is very important that the lines are correctly treated, that dressings are changed when needed and that they are properly marked so the next nurse knows to change it on that day, in other words to follow protocols and if I have that prioritise an antibiotic or cure a little ulcer here, the antibiotic will come first. If we are advancing and the situation changes, my priority may be this silly little ulcer and the antibiotic can be given later and nobody will say, 'You haven't signed it and it is one o'clock,' I'm tending to the wound,' nobody is going to say I shouldn't, I'm tending to it so it doesn't get worse, so that it doesn't cause discomfort…, your priorities gradually change”, (*N2/ YE: 20)

Something that concerns and mobilises the nurses in defence of the patient is the prevention of what they consider practices that go beyond the reasonable treatments that impede a dignified death.

*“…the complications started… what were they doing? …tests and more tests… sometimes there are confrontations between the team, especially between doctors and nurses, because what are we doing, right? …Before that process of… accompanying her and letting her die was harder…”* (N3/ YE: 20)

Sometimes they are truly heroic acts that reveal the confrontation and lack of equality in the relationships within the team. In the following story the nurse tells of how she strives to actively defend a vulnerable patient to be able to present an accurate image to the family and allow dignified death:

“… we were doing rounds and I went and I explained… And then the doctor dealing with her, I spoke to him first, 'listen, do you think this makes sense? Have you ever seen a sepsis with a CID in this state get better? I haven't.' 'Ah well, you never know'. What do you mean, you never know!… I went to the rounds and I explained… everybody agreed that there was no way out, everybody except him, and he decided he had to speak with the family and propose palliative care… the last word belongs to his doctor (said the supervisor”, (N2/ YE: 20)

The nurses consider it important to understand the expectations, desires and wishes of the patient and interpret that the family is important for comforting patients in palliative care. Based on this idea, as seen in the following extracts, they recognise that ensuring the family is present is a responsibility that is part of their role, a decision that is part of the care of the patient:

*“…there was no solution, and we left the family with her so she didn't feel alone… In these situations … I think it is the family is necessary so they feel all right…”* (N 7/ YE: 21). *“…it was palliative care so they were allowed to be there longer than normal. It was agreed that, in each shift if the nurse felt it was appropriate the family could stay longer, I felt it was a good idea and so they stayed with her” (*N2/ YE: 20). *“…The day she died her husband was there all day and instead of keeping strict visiting hours, they were extended…”* (N 10/ YE: 10). *“…so that the woman…, taking into account her suffering, could experience the situation and get used to the idea”* (N 13/ YE: 7).

It is a knowledge in which the nurses with experience are able to make qualitative distinctions in relation to the position of the families. As in the following story, where the nurse distinguishes the different needs of two families:

*“…a man who came in one day and died the next, it was a very acute process, where there was nothing left to do… and the family asked for treatment to be limited, he was terminal and there was nothing left to do and the family said they didn't want more machines and other things… and we asked them if they wanted to go in and they said no, they would rather remember him as he was… In contrast, the other family, I remember that I got to work one night, and he was about to die, and the family had been there all afternoon coming and going and at 10.30 pm we saw it was coming to an end so we told the family to go in and they were very touched because they didn't expect it…”,* (N 7/YE: 21).

Something that particularly concerns these nurses is how difficult it is to for someone to come to terms with an irreversible or extremely serious situation of their relative, and which leads some families to “hear what they want to hear” when given medical information. In the following story we can see how the nurse, driven by compassion, tries to adopt a position of candour with the family to help them come to terms with the patient's transition. The story does not show an prior analysis of how to help the families face the impact of "a truth" they do not want to hear:

*“… what experience tells me is that sometimes you have to be a bit cruel and say the word die, because sometimes when you say he's very bad to someone, their very bad isn't the same as yours, 'it's very serious,' fine, but that's why he's here, they don't understand it never happens to me, but even I'd end up blind to it, I wouldn't see him and say, 'well he's bad but he's here, no,' so I believe there you have to be a but cruel in that sense, well you know that the doctor's said it to them, you've even been there and heard exactly what was said, so I believe you can use those words 'the doctor's told you that Teresa (not her real name) is in a process.' But not like that, clearly, 'he's in a process that is irreversible…' when you see that they're looking for words from you that are the opposite of what they've heard, because that's not what they want to hear, patients who have had strokes and have no possibility of recovery and they come into the room and say, 'so how did he get through the night, good, right?', but what does getting through the night mean in his state, right? And so you have to say, 'well, what did the doctor say to you in rounds? He told you the condition this person is in, yes… or no'”,* (N 2/ YE: 20).

This issue takes up a significant part of stories of the nurses that deal with it. Two aspects that are central to their discourse as a guideline for this practice: their involvement in achieving a dignified death for the patient and their compassion for the family's suffering.

### How to protect and defend the patient from errors

Three of the more experienced nurses told us of the knowledge and characteristics that sustain this practice. One of these aspects is the knowledge they have of the team members and the relationship with them. It is knowledge that the nurses use to organise and ensure the care practices. And something that is shown as a characteristic of the more experienced nurses is that when establishing care priorities they take into account the level of competence of the team members and the risk and severity of the patients. As the next story illustrates, the nurse provides several examples of how she reorganises the patient allocation and takes responsibility for their care when she sees they are agitated or when their assisted respiration tube is going to be removed. She even shows signs of being conscious of the reassurance that she brings to her less experienced colleague and the benefit for the patient (less risk and a higher chance of success) that an experienced nurse entails. In this story we can also see she is interested in knowing her colleagues and is able to recognise the limits of their competence. Knowledge that is only used to benefit the patient, the less experienced professional and we suppose, for her own peace of mind:

*“…When a patient on the unit is disorientated or agitated, I always take them, because of my years of experience and because I am able to calm them… I have my colleagues take my patients that need less attention and I apply the law of priorities… I delegate among my colleagues, they have always helped me… if the process of withdrawing ventilation is done correctly it is a success for the patient and for the professional, because it avoids more days in hospital, aftereffects for the patient; it's very important to me to do it well… I work with a lot of young people and you see how they acquire this intuitive intelligence… you see how they are concerned about the patient, if something is happening to them, they tell me they don't know what's happening with that patient, but something's going on, so we go and see what's happening.”,* (N 6/YE: 29)

Another thing that also stands out is in this story the nurses' commitment to the new nurses' learning. We can see how the nurse recognises the lack of care by a new nurse, who labels a patient as “hysterical” and confronts her with an incident that makes her reflect, and she is conscious that this confrontation can be useful as an experience. An action that only seeks to prevent the abuse that is the new nurses labelling patients and to help them gain experience:

*“…I am finding young people that judge directly: this one's “AIDS-infested”… an example from 48 hours ago, a nurse that graduated in the last academic year does the handover for an 18-year-old patient and says she's “hysterical”, and I answered that people aren't sent to the ICU for being hysterical, this person is in here with pneumonia. That same night the patient ended up intubated. The next day that nurse said to me, “You know what, nobody has ever said anything like that to me before. When I saw her just now it stopped me in my tracks; I have to work on that.” That fact will serve as an experience for that nurse…”,* (N 6/ YE: 29)

In the following story, the nurse also shows knowledge of the team which enables her to distinguish the ingrained prejudices that can condition good practices. The nurse knows through experience the prejudices that the team members (nurses and doctors) may have towards an ethnic gypsy family and describes how she organises the relationships so that the mother of a young patient could be with him and help his recovery:

*“… I saw that (the mother?) knew what she was talking about, and with so much enthusiasm, she wouldn't give up… I spoke to Dr.…, this time I did consult more people in the team because they were gypsies. It's a problem, you let the mother in and 30 more come in, into a unit where everyone can see… 'There are 30 in there and they won't let me in.' It was a problem. … I said to him, 'What do you think? It looks to me like this girl is going to behave properly and respect the regulations because she's going to see that it's a big thing that we let her in.' We arranged that I would speak to her and make an agreement…and she was the best mother in the world”,* (N 2/YE: 20).

Another aspect where we can differentiate the knowledge of these nurses is in how attentive they are to the action of young doctors in terms of preventing errors. The nurse in the following extract recounts the significance that it has for her to maintain a good relationship and that these doctors maintain the perception of control over their work. To preserve this aspect the nurse appears to have interiorised (or ritualised) established ways of solving the problem, as seen in the story where the nurse tells us in detail about the strategy created to avoid confrontations in the prevention of medical errors:

*“… When I see something that isn't right, I know not to go and say 'look, you've made a mistake,' no, that's already a confrontation… So it's a case of seeing what he wanted to put,… 'Look, I've been reading this and I'm not sure if you meant to put this or if you didn't realise and you've put that,' and then I make him go back and read it again. I tell them, 'normally the guideline says one gram every six hours and as you've put down two I don't know if you've done it for a reason, because you have your opinion, or because you didn't realise and put one instead of two… Look, check it with your supervisor, but I think it's one,' so he then goes and checks with his supervisor and I haven't confronted him at all and I've helped him and at the same time the patient”* (N 2/ YE: 20).

In their stories, these nurses also speak of how they protect and defend their patients that they undertake as an ethical commitment to the patient, and sometimes with a certain fear of conflict with the power of the other. These practices are linked to situations in which the nurses believe the patient is or may be harmed by treatment they consider inadequate. These practices for the protection and defence of a patient are knowledge that the experienced nurses base on knowledge of the patients that enables them to anticipate their responses to the treatments and mobilise them firmly and safely to protect them from error. These nurses, who are aware that their experience will generally be “heard”, help the doctors make decisions, or even take some responsibility in the diagnosis and treatment:

*“…The doctors wanted to limit the therapy and I felt it was too soon… she was 20 years old … the doctor on the night shift said to me, 'She'll die tonight. Do what you want because she won't get through the night,' and I really did what I thought was right… the night shift doctor said, 'If you'll feel better about it, do what you want. You can increase or decrease her medication as you wish.' …, and they adjusted the medication to what I decided and the next day she was still alive. They adjusted the treatment again and the girl improved and started to pull through. It took days but she pulled through…”,* (N 6/ YE: 29). *“… sometimes they say, 'extubate him' and you say to them. 'I still don't think he's ready to be extubated,' because he doesn't cough, he doesn't make the effort, he's very weak… and the doctors do pay attention to us… generally they pay a lot of attention!”, (*N 7/ YE: 21).

This issue and its practice show a link to the knowledge and empowerment with the experience accumulated over years of work and with the involvement with and commitment to the patients. These characteristics are discovered as components important of caregiving that prevent errors and maintain the relationships of team for the benefit of the patient.

## Discussion

Our results revealed *knowledge* that the ICU nurses in this study execute on a daily basis to cover critical moment in the care of the patient. The more experienced nurses are very detailed in their description of how they work in particular situations and moments. They are practices that approaches the reflective practice described by Schön [20], which links the art of practice with research, and show the narrative compression of the nurses in the detail they use when narrating as the patient's condition changes [1,4].

The *know-how* of the more experienced nurses is based on the ability they show to perceive reality they are alert to note the smallest of details, that show a special sensitivity for detecting emotional concerns and in the intuitive recognition they have of the situation [6,21,33]. The nurses with more experience in terms of years also had the greatest ability to take advantage of intuitive and analytical aspects of decision-making, recognising the most relevant clinical signs and aspects in each situation, identifying the actions that were necessary and their possible results and reorganising their participation in patient care [34].

The practice ***Connect with, calm and situate patients who cannot communicate*** is a sensitive practice, in which the nurses strive to make the patients understand their situation, using humour and with emotional involvement that can be linked to the management and construction of knowledge described by Inger, Andershed Gustavsson and Terneste [4]. In the calmness and the time devoted to understanding the patient, it can be seen that they wish to transmit to the patient the sensation of being there that was described by Benner and Wrubel as a characteristic of expert nurses [35].

In the practice ***Situate and provide relief to patients in transitions of mechanical respiration***, one aspect that we feel is very important is this nurse's statement that success in weaning largely depends on what the professional is like; if the nurse is distracted by other things or has too much work it is more likely that the weaning will fail, with *everything* that this entails for the recovery of the patient. We wondered whether this, which seems to be part of the *culture shared* by all of the professionals, is taken into account when scheduling and performing the *withdrawal of mechanical ventilation* from the patients. Although we have not found any explicit references to this aspect, various authors conclude that a collaborative task prepared in advance and the autonomy of the nursing decisions provide the best results in weaning [36-38]. The predictive factor of the nurses' judgement also appears to be clear in the result of weaning [39]. We are in all cases faced with an ethical dimension of the caregiving practices that may be learned by others in this narrative *know-how*.

In patients with non-invasive ventilation the nurses explain some of the main causes that can lead to the failure of the treatment described in previous studies, such as oppressive and badly fitting masks, a lack of patient-ventilator synchronisation or inappropriate practices [40,41]. The nurses gave specific details of how to overcome these difficulties and respond with attention to the perceptions of the patients, remaining relaxed and communicating calmly to help them restore control of their breathing. Some of these caretaking components were demonstrated in the study by Sørensen et al. [42].

The findings in practice on ***How to provide reassurance and guarantee the safety of immobilised patients*** illustrate the wisdom the experienced nurses have acquired in reading the situation of the patient. It is knowledge that they show in the way they manage their fears, situating them to make them aware of their situation and responding calmly with a story that aims to comfort the patients and transmit a sensation of control. A significant finding is in the reading they make of immobilised patients with physical restraints and how they show themselves to be responsible for deciding when and how to use the restraints. Unlike other studies with senior in-patients [43] the absence of negative feelings in the ICU nurses in this study suggest that the concern for guaranteeing the critical patient's safety is what is responsible for the nurses experiencing this treatment as a defence of the patient. The aspects that they evaluate in the use of physical restraint, as was described in the study by Choi and Song [44], are agitation and prevention of the extraction of mechanical devices. Although it is clear that there are ethical considerations regarding its use in the commitment that they take on with the patient to remove the restraints when they feel the risk has been surmounted, there is neither the dilemma [45] nor the possibility of treating them with other means. They do not seem to agree, as indicated by Hine [46], that physical restraints can increase agitation and can have devastating physical and psychological effects on the patient in addition to doubtful benefits [46-49]. The use of physical restraints is still an unsolved problem in clinical practice. These findings suggest that the experienced nurses in the ICU have areas that have barely been explored that can impede a culture of alternative action and the need for training [50], but they also suggest the need to research why the experienced nurses continue without considering other practices alternative to restraints.

Regarding the practice ***The “connection” with patient in coma***, the nurses in this study, like in the study by Baker [51], state that verbal communication with unconscious patients is something they do and consider important, and although they are not always sure that the patient can hear them they do it “just in case”. This statement leads us to believe that is knowledge incorporated in a shared, timid culture that knows, sometimes not completely consciously, things for which there is still not enough “scientific evidence” although different studies [52,53] and neuroscientists such as Owen are pointing out the possibility that people in comas can establish some degree of connection [54,55].

In the practice, ***Taking care of the body*** is the type of knowledge that regulates the nursing activity and constitutes know-how that is consolidated in a number of skills that are constituted as a result of experience [6], of reasoning during the action [20] and involvement [18]. It is knowledge that the expert nurses have and that is seen in the meticulousness and beauty with which the experienced nurses describe every detail of what they do. The way they make the bed when the patients cannot be moved, making sure they do not leave any wrinkles that could damage their skin, the way they wash them so they are comfortable and their intracranial pressure does not increase or how they alleviate the patients' perception of fear if they think they are about to experience added harm are some of the issues that stand out in this knowledge. The importance of these practices for the experienced nurses indicates that taking care of the body and preventing pain, fear and the risk of injury that the routine care entails implies that the nurses have must recognise them [56].

In ***The transition of care from saving life to palliative care*** among the nurses in our study there is a consensus in aiming to achieve a *dignified death* in which they prioritise the comfort of the patient, that they do not die alone and the possibility of saying goodbye to their family. And there is conflict when they feel that the transition to palliative care is being delayed with a use of medical treatment and technology beyond what is reasonable or when they do not present a realistic picture to the family that will enable them to understand the patient's situation and make decisions. These results confirm the findings of other studies [57-62] and, like in the study by Beckstrand et al. [60], prolonging the death of a patient, when it is inevitable, causes the nurses moral disquiet. Nevertheless the nurses in our study with the most years of experience channel this distress into involving themselves in the defence of the patient. Although some doctors seem to be insensitive to the suffering and impervious to the opinions of the nurses, this committed attitude of these nurses seeking, not confrontation with the doctors, but rather consensus to reorient attention to palliative care illustrates a strategy that has already been put forward by Bardger [63]. This commitment and active role also leads these nurses to help to present the families with a realistic image of what is happening, aware that the family may be in denial and that it may be difficult for them to understand and accept the medical information when the patient is dying.

In the practice ***How to protect and defend the patient from errors*** the nurses with the most years of experience demonstrate how they know the members of the team and how they use this knowledge to organise care practices and avoid errors. They are differentiated by the commitment they assume with the patients and the attentive and careful way in which they secure the learning of new nurses and doctors. This responsible way of acting with the other team members was indicated by Benner, Tanner and Chesla [7] as a moral art of the expert nurses. Knowledge and good judgment of the clinical situation of the patients that enables them to be in a “strong” position when faced with doctors or other nurses to achieve what they consider the patients need. A practice that they undertake because *they feel* obliged to act when they believe that the medical treatment or the intervention of another nurse is inappropriate for the patient. They approach these actions, as indicated [7], with a prudent attitude and a realistic vision of the possibilities, limits and capabilities of the others.

The knowledge and practices of the nurses of this study suggest, as indicated by Gail [14], that the nurses are refocusing their activity on the organisation of a more ethical basis for the praxis, that arises from the nursing philosophy and that is co-constituted with the patients and the group. In addition they describe, as Inger [4] believes, the hermeneutic construction of nursing knowledge as a journey that travels through sensitivity, emotional involvement, comprehension of the patient's world and attention to the context.

## Conclusions

The stories of experiences describe a contextual practical knowledge that the more experienced nurses develop as a natural and spontaneous response.

The narrative based on evidence enables us to clearly capture the knowledge of nursing practice, especially in aspects as complex as the care involved in human relations and that are difficult to articulate in merely technical discourse.

In critical patients the application of everyday practical knowledge greatly influences their well-being. In the cases in which the nurses describe how they have protected the patients from an error can mean the difference between the life and death. This idea suggests that the autonomy of the experienced nurses must be accepted as a key practical principle.

Knowledge plays a significant role in the innovation and development and in this task the results of this study indicate the need to manage tacit knowledge, focusing on the professional, on communication, to improve the transfer of knowledge and collaboration.

### Key messages

• It is important to acknowledge that in practice knowledge is developed that must be explored.

• Nursing knowledge regarding the fears of the patient and *knowing* how to act to achieve the greatest emotional well-being increases the sensation of control of the patients.

• Taking care of the body is knowledge that experienced nurses demonstrate in the meticulousness and beauty with which they describe every detail of what they do.

• The knowledge and committed attitude of the experienced nurses help the patient have a dignified death with their family by their side.

• The knowledge that the experienced nurses have of the members of the team enables them to organise care practices, avoid errors and contribute to the learning of the new staff.

## Abbreviations

ICU: Intensive care unit; ICP: Intracranial pressure.

## Competing interests

The authors declare that they have no competing interests.

## Authors’ contributions

MSAU conceived and designed the study, acquired the study data, analyzed and interpreted the study data, and wrote the manuscript. JLMM and CFG participated in the analysis and interpretation of the study data and contributed important intellectual content to the final version of the manuscript. All the authors have read and approved the final manuscript.

## Pre-publication history

The pre-publication history for this paper can be accessed here:

http://www.biomedcentral.com/1472-6920/14/173/prepub
